# Analysis of *EIF4G1* in ethnic Chinese

**DOI:** 10.1186/1471-2377-13-38

**Published:** 2013-04-26

**Authors:** Kai Li, Bei-sha Tang, Ji-feng Guo, Ming-xing Lou, Zhan-yun Lv, Zhen-hua Liu, Yun Tian, Cheng-yuan Song, Kun Xia, Xin-xiang Yan

**Affiliations:** 1 Department of Neurology, Xiangya Hospital, Central south university, xiangya road, Changsha, China; 2 Neurodegenerative Disorders Research Centrer, Central South University, xiangya road, Changsha, China; 3 National Lab of Medical Genetics of China, xiangya road, Changsha, China

**Keywords:** *EIF4G1*, Parkinson’s disease, Ethnic Chinese

## Abstract

**Background:**

Eukaryotic translation initiation factor 4-gamma 1 (*EIF4G1*) gene mutations have recently been reported in autosomal dominant, late-onset Parkinson’s disease (LOPD). We carried out genetic analysis to determine the prevalence of *EIF4G1* variants in an ethnic Chinese population and to better understand the association between *EIF4G1* and PD.

**Methods:**

We conducted a comprehensive genetic analysis of *EIF4G1* in a cohort of 29 probands of autosomal dominant, LOPD families. Polymerase chain reaction (PCR) analysis and sequencing was carried out of the entire *EIF4G1* exonic regions and exon-intron boundaries. Specific mutation and exonic variants were chosen for further sequencing in a case–control study including 503 sporadic PD and 508 healthy controls. Statistical significance was analyzed by the Chi-square test.

**Results:**

Our analysis revealed three exonic variants (rs2230571, rs13319149 and rs2178403) and eight intronic variants across the entire *EIF4G1* gene. No reported mutations were detected in *EIF4G1* exonic regions. The synonymous coding variant rs2230571 in exon 27 and the eight intronic variants were not used for further sequencing, but the specific mutation c.3614G > A (p.R1205H) and the two nonsynonymous variants (rs13319149 and rs2178403) were chosen for further analysis in a case–control study. None of the 503 sporadic PD or 508 healthy controls carried p.R1205H, and there was no statistical significance in rs2178403 genotype or allele frequencies in *EIF4G1* between the PD cases and the healthy controls (*p* = 0.184 and *p* = 0.774, respectively; Chi-square test). The rs13319149 genotype in all PD cases and healthy controls was GG.

**Conclusions:**

Our data indicate that in an ethnic Chinese population, the pathogenic mutation p.R1205H in *EIF4G1* is not common and that *EIF4G1* exonic variants rs2178403 and rs13319149 are not associated with PD. *EIF4G1* does not appear to be a frequent cause of PD in this ethnic Chinese population.

## Background

Parkinson’s disease (PD) is the second most common neurodegenerative movement disorder and is clinically characterized by the cardinal features of bradykinesia, resting tremor, rigidity, postural instability and responsiveness to dopaminergic therapy. Most PD patients are sporadic but approximately 5–10% are familial [1]. Although the etiology of PD still remains elusive, certain genes are known to influence PD susceptibility, for example, autosomal dominant PD is associated with *SNCA*[2], *LRRK2*[3], *UCHL1*[4] and *VPS35*[5,6], recessive parkinsonism with *PRKN*[7]*, PINK1*[8] and *DJ-1*[9], and more complex forms of recessive parkinsonism with *ATP13A2*[10], *PLA2G6*[11] and *FBXO7*[12]. Several techniques have been used to identify candidate genes in familial PD, including classic linkage analysis, positional cloning studies and recent exome sequencing [2,5,6,13].

Recently, Chartier-Harlin et al. [14,15] performed genome-wide linkage analysis of a multi-incident French family presenting with autosomal dominant, late-onset PD (LOPD). They found that the mutation c.3614G > A (p.R1205H) in *EIF4G1*, encoding eukaryotic translation initiation factor 4G1 (eIF4G1), a component of the translation initiation complex eukaryotic translation initiation factor 4F (eIF4F), caused the familial LOPD. The translation initiation complex is a large family [16,17], including eukaryotic translation initiation factor 4E (eIF4E) and eukaryotic translation initiation factor 3e (eIF3e) that interact with each other and conduct normal translation initiation activity [14,18,19]. In the Chartier-Harlin study [14], it was predicted that mutations p.A502V and p.R1205H disrupt eIF4G1 binding to eIF4E or eIF3e, resulting in PD. Subsequent sequencing and genotyping analysis also identified different *EIF4G1* mutations in affected subjects with familial parkinsonism and idiopathic Lewy body disease in different populations.

To obtain a comprehensive view of the prevalence of *EIF4G1* variants in an ethnic Chinese population, we conducted a comprehensive genetic screening of *EIF4G1* in a Han Chinese group of subjects.

## Methods

### Patients

The study comprised 29 probands of PD families compatible with autosomal dominant inheritance (two or more PD cases in at least two consecutive generations) and 503 sporadic PD patients of Chinese Han ethnicity from Hunan, Hubei, Jiangxi, Sichuan and Chongqing provinces of China. All patients were consecutively recruited from the Department of Neurology of Xiangya Hospital (Central South University, State Key Laboratory of Medical Genetics of China) and the Neurodegenerative Disorders Research Center. A standard clinical neurological examination was performed for all patients by two neurologists, and a diagnosis of idiopathic PD was made according to the United Kingdom PD Brain Bank Criteria [20].

There were 13 male and 16 female PD patients among the 29 autosomal dominant, LOPD probands, with a mean age at onset of 50.33 ± 9.56 years (range, 41–63 years). Of the 503 sporadic PD patients, 269 were male and 234 were female with a mean age at onset of 55.25 ± 12.21 years (range, 22–81 years). The case–control study also included 508 healthy controls (268 males and 240 females; average age at examination, 56.95 ± 15.20 years, range, 25–88 years) matched for age, gender, ethnicity and area of residence. The control subjects were healthy people from the Xiangya Hospital Medical Center, and healthy volunteers with no history of neurodegenerative diseases. A standard clinical neurological examination was performed on all control subjects, and a diagnosis of possible idiopathic PD was excluded according to the United Kingdom PD Brain Bank Criteria. There was no statistically significant difference in age or gender between patients and controls (*p* > 0.05, using Chi-square test). The study protocol was approved by the Ethics Committee of Central South University and written informed consent was obtained from all patients and controls.

### Genetic analysis

Genomic DNA was extracted from peripheral blood using standard protocols. Polymerase chain reaction (PCR) analysis was carried out using 20 primer pairs (Additional file 1: Table S1, available online), and each PCR product was purified and directly sequenced in both forward and reverse directions on an ABI 3100 automated sequencer (Applied Biosystems, Foster City, CA). We initially sequenced the entire *EIF4G1* exonic regions and exon-intron boundaries of the 29 probands of autosomal dominant, LOPD. Sequence alignment and analysis were carried out with Chromas 2.12 software (Technelysium Pty, Inc., Queensland, Australia).

### Statistical analysis

Allele and genotype frequencies of PD and control groups were analyzed using the Chi-square test with SPSS 17.0 software (SPSS Inc., Chicago, IL, USA). A two-tailed test *p*-value of <0.05 was considered statistically significant.

## Results

We identified three exonic variants and eight intronic variants across the entire *EIF4G1* gene (Table 1). No reported mutations were detected in the *EIF4G1* exonic regions in any of the 29 probands. One of the three exonic variants (exon 25, rs2230571) was synonymous, the other two (rs13319149 and rs2178403) were nonsynonymous.

**Table 1:** ***EIF4G1 *gene variants in 29 probands of LOPD families**

**Exonic/intronic variants**		**Accession number**	**Nucleotide change**^**a**^	**Amino acid change**	**Frequency of variant in LOPD families (%)**
1	Intron 2	rs73187631	IVS2-182 a > t		7.41
2	Intron 3	novel	IVS3 -44 a > g		1.85
3	Intron 4	novel	IVS4 + 115 g > a		3.70
4	Exon 7	rs13319149	c.481A > G	T161A	100
5	Intron 9	rs9846954	IVS9 + 93 a > t		25.93
6	Intron 9	novel	IVS9 + 75 indel^b^		7.41
7	Intron 9	rs4912537	IVS9-100 c > t		14.81
8	Exon 10	rs2178403	c.1294A > G	M432V	55.56
9	Intron 15	1KG 3 184041398	IVS15 + 17 c > t		1.85
10	Intron 18	1KG 3 184043002	IVS18 + 100 g > t		1.85
11	Exon 27	rs2230571	c.4005C > T	H1335H	18.52

^a^ Human *EIF4G1* cDNA sequence (RefSeq Accession number NM_198241.2) was used as the reference sequence.

^b^ indel : acttgaac deletion; tgggtaccagagaa insertion. (i.e. acttgaac > ggtaccagagaa).

Neither the synonymous variant nor the eight intronic variants could be sequenced, but the specific mutation p.R1205H and the two nonsynonymous variants underwent sequencing analysis in the 503 sporadic PD patients and 508 controls. None of the patients or controls carried the specific mutation p.R1205H. The nonsynonymous exonic variant rs2178403 was in Hardy-Weinberg equilibrium in both the patient and control groups using a goodness-of-fit Chi-square test. Genotype and allele frequencies of rs2178403 for all subjects are shown in Table 2. No statistically significant differences were observed regarding rs2178403 genotype or allele frequency between patients and controls (*p* = 0.184 and *p* = 0.774, respectively). Regarding the nonsynonymous exonic variant rs13319149, all patient and control genotypes were GG.

**Table 2:** Genotype and allele frequencies of rs2178403 in PD and controls in Han Chinese

**N**	**Genotype (%)**				**Allele (%)**		
	**AA**	**AG**	**GG**	**P value(df )**	**A**	**G**	**P value (df , OR)**
Patients 503	74(14.7)	226(44.9)	203(40.4)	0.184^a^ (1)	374(37.2)	632(62.8)	0.774^b^ (1, 1.027)
Controls 508	87 (17.1)	210(41.4)	211(41.5)		384(37.8)	632(62.2)	

P Values after correcting by binary logistic regression with age and gender using SPSS 17.0: ^a^ p = 0.713, ^b^p = 0.702.

## Discussion

*EIF4G1* was confirmed as a candidate PD gene by Chartier-Harlin et al. [14,15], but recently three other groups identified *EIF4G1* mutations in both PD patients and healthy controls [21-23]. This raises questions about the causality of the gene with regard to PD. In our comprehensive genetic analysis of *EIF4G1*, we also obtained similar negative results. The specific mutation p.R1205H, first identified by Chartier-Harlin, is important in PD for regulating cell survival in response to stressors [14,15]. However, it was absent from all of our 503 sporadic PD cases and 508 healthy controls suggesting that different populations may have different pathogenic mutations. Alternatively, the effect of the same mutation may differ in different populations, as indicated by the presence of mutation p.R1205H in control individuals of a Central European population shown by Schulte et al. [23].

In the present study, exonic variants rs13319149 and rs2178403 showed no significant difference in either genotype or allele frequencies between PD patients and controls, suggesting that they are probably benign polymorphisms. Although *EIF4G1* is considered a pathogenic gene of LOPD, our 29 probands were not typical of LOPD as the average age of onset was 50.33 ± 9.56 years. This may contribute to our negative results, together with the fact that our sample size was relatively small. Further larger-scale genetic studies should therefore be performed to validate our findings.

## Conclusions

To our knowledge, this is the first study to assess the frequency of *EIF4G1* variants in a cohort of Chinese PD patients and controls. Our results suggest that in this ethnic Chinese population, the pathogenic mutation p.R1205H in *EIF4G1* is not common and that nonsynonymous exonic variants rs2178403 and rs13319149 are not associated with PD. Thus, it appears that *EIF4G1* is not a frequent cause of PD in this ethnic Chinese population. Further large scale genetic and functional studies would be useful to establish *EIF4G1* associations with PD.

## Abbreviations

ATP13A2: ATPase type 13A2 gene; df: Degree of freedom; eIF3e: Eukaryotic translation initiation factor 3e; eIF4E: Eukaryotic translation initiation factor 4E; EIF4G1: Eukaryotic translation initiation factor 4-gamma 1 gene; FBXO7: F-box protein 7; LOPD: Late-onset Parkinson disease; LRRK2: Leucine-rich repeat kinase 2 gene; OR: Odds ratio; PCR: Polymerase chain reaction; PD: Parkinson disease; PINK1: PTEN-induced putative kinase 1 gene; PRKN: Parkin; PLA2G6: Phospholipase A2, group VI gene; SNCA: A-synuclein gene; UCHL1: Ubiquitin carboxyl-terminal esterase L1; VPS35: Vacuolar protein sorting 35 gene

## Competing interests

The authors declare that they have no competing interests.

## Authors’ contributions

KL performed the genotyping, statistical analysis, and drafted the manuscript. MXL, ZYL, ZHL, YT, CYS, and KX contributed to the collection of materials, participated in the study design and coordination, and drafted the manuscript. BST, JFG and XXY conceived the study, participated in its conceptual design and coordination, and drafted the manuscript. All authors read and approved the final manuscript.

## Pre-publication history

The pre-publication history for this paper can be accessed here:

http://www.biomedcentral.com/1471-2377/13/38/prepub

## Supplementary Material

Additional file 1: Table S1 *EIF4G1* Sequencing Primers. Click here for file
