# Supplementary material for: Analysis of EIF4G1 in ethnic Chinese
Source: BMC Neurol. 2013 Apr 26;13:38. doi: 10.1186/1471-2377-13-38 (PMC3663786; doi:10.1186/1471-2377-13-38)
Supplement: Additional file 1: Table S1 — EIF4G1 Sequencing Primers. [file 1471-2377-13-38-S1.docx]

Additional file 1: Table S1. *EIF4G1* Sequencing Primers

| Exon | | Forward Primer | Reverse Primer |
| --- | --- | --- | --- |
| E1 |  | GGGCGGGCTGTGGCTGTCAC | GGGCGGGCTGTGGCTGTCAC |
| E2+3 |  | CAGCTAGCTCCGTTCGTGAT | CTGGTGAAATAAGGGGAGCA |
| E4 |  | GGAGGGGGTATGTGGATTGT | CAATAGCAGCCTCCGGTAAA |
| E5+6  E7  E8+9  E10a  E10b  E11+12+13  E14+15  E16+17  E18+19  E20+21  E22+23  E24  E25+26  E27+28  E29+30  E31+32  E33 |  | [GCCATTTGGTCAAGTGGTCT](http://browser.1000genomes.org/Homo_sapiens/Variation/Mappings?db=core;g=ENSG00000114867;r=3:184032283-184053146;v=rs13319149;vf=18031257;source=dbSNP)  GGTGTCAGGCAGGCATTAGT  TGGCACCTACCTACCTGCTT  [TGAGGTGGTGGAGTGACTTG](http://browser.1000genomes.org/Homo_sapiens/Variation/Mappings?db=core;g=ENSG00000114867;r=3:184032283-184053146;v=rs4912537;vf=17666183;source=dbSNP)  [TGAAACTGGGGAGCCATATC](http://browser.1000genomes.org/Homo_sapiens/Variation/Mappings?db=core;g=ENSG00000114867;r=3:184032283-184053146;v=rs2178403;vf=17568754;source=dbSNP)  [GGTGATGCAAAGGGGAAATA](http://browser.1000genomes.org/Homo_sapiens/Variation/Mappings?db=core;g=ENSG00000114867;r=3:184032283-184053146;v=1KG_3_184041398;vf=43776730;source=1KG)  [GAGCCTGAGGTCCTGAAAGA](http://browser.1000genomes.org/Homo_sapiens/Variation/Mappings?db=core;g=ENSG00000114867;r=3:184032283-184053146;v=1KG_3_184043002;vf=43776743;source=1KG)  [TGTGCCCTCTTTGCTTCTTT](http://browser.1000genomes.org/Homo_sapiens/Variation/Mappings?db=core;g=ENSG00000114867;r=3:184032283-184053146;v=rs2230571;vf=17570997;source=dbSNP)  TAGCCGAGTGGCTGGTTATC  GGTGTGTGTCCCCCTCCT  CTGGGCCATTCACTACCTGT  TTGGGTTAGATTGGGGCATA  GGAGAGGAATGGAGGGAAAG  GTTCCTGGGGGTTCCATAGT  CTGCACCAGACCGTAGGAAT  TGAAGGAGGGCAGCAGTAAC  ACTCTGGAATGGCCACAAAT | GCTGGTGCCTGATCCTACAT  GGCCACAGAAAACAAACCAT  TAACCCCGCATCATCTTCTC  CCACTGTGTGAGATGCCAAA  AGGGACCCAGAAACATGTCA  GCAAAGAGGACAGGAAAAGG  GGGGACTTAGGCATGACAGA  CGTCCTGAGCCAGAGAAATC  AATGGTCTTGGGACCCTGAT  TCTCCAGCAATCCAACCAGT  TTGGGCCTTGTTCTTAGCAT  TCACTGTGGGAAGAGAACACA  CTCCCAAAGTGCAGGGATTA  CCCAGGTACCAACACAATCC  AATTGGGCCCTTCAGTCTTC  TTCCATGAAGGGTCTCATGC  TCTAGAGGGAGCAGGGCATA |
